# Additive effects of *LPL*, *APOA5 *and *APOE *variant combinations on triglyceride levels and hypertriglyceridemia: results of the ICARIA genetic sub-study

**DOI:** 10.1186/1471-2350-11-66

**Published:** 2010-04-29

**Authors:** María-José Ariza, Miguel-Ángel Sánchez-Chaparro, Francisco-Javier Barón, Ana-María Hornos, Eva Calvo-Bonacho, José Rioja, Pedro Valdivielso, José-Antonio Gelpi, Pedro González-Santos

**Affiliations:** 1 Departamento de Medicina y Dermatología, Facultad de Medicina, Laboratorio de Lípidos y Arteriosclerosis, Centro de Investigaciones Médico-Sanitarias (CIMES), Universidad de Málaga, Campus de Teatinos, 29010 Málaga, Spain; 2 Ibermutuamur Cardiovascular Risk Assessment (ICARIA) Study Group, Ibermutuamur: Mutua de Accidentes de Trabajo y Enfermedades Profesionales de la Seguridad Social n° 274, 28043 Madrid, Spain; 3 Departamento of Medicina Interna, Hospital Universitario Virgen de la Victoria, Campus de Teatinos, 29010 Málaga, Spain; 4 Departamento de Bioestadística, Facultad de Medicina, Universidad de Málaga, Campus de Teatinos, 29010 Málaga, Spain

## Abstract

**Background:**

Hypertriglyceridemia (HTG) is a well-established independent risk factor for cardiovascular disease and the influence of several genetic variants in genes related with triglyceride (TG) metabolism has been described, including *LPL*, *APOA5 *and *APOE*. The combined analysis of these polymorphisms could produce clinically meaningful complementary information.

**Methods:**

A subgroup of the ICARIA study comprising 1825 Spanish subjects (80% men, mean age 36 years) was genotyped for the *LPL*-HindIII (rs320), S447X (rs328), D9N (rs1801177) and N291S (rs268) polymorphisms, the *APOA5*-S19W (rs3135506) and -1131T/C (rs662799) variants, and the *APOE *polymorphism (rs429358; rs7412) using PCR and restriction analysis and TaqMan assays. We used regression analyses to examine their combined effects on TG levels (with the log-transformed variable) and the association of variant combinations with TG levels and hypertriglyceridemia (TG ≥ 1.69 mmol/L), including the covariates: gender, age, waist circumference, blood glucose, blood pressure, smoking and alcohol consumption.

**Results:**

We found a significant lowering effect of the *LPL*-HindIII and S447X polymorphisms (*p *< 0.0001). In addition, the D9N, N291S, S19W and -1131T/C variants and the *APOE*-ε4 allele were significantly associated with an independent additive TG-raising effect (*p *< 0.05, *p *< 0.01, *p *< 0.001, *p *< 0.0001 and *p *< 0.001, respectively). Grouping individuals according to the presence of TG-lowering or TG-raising polymorphisms showed significant differences in TG levels (*p *< 0.0001), with the lowest levels exhibited by carriers of two lowering variants (10.2% reduction in TG geometric mean with respect to individuals who were homozygous for the frequent alleles of all the variants), and the highest levels in carriers of raising combinations (25.1% mean TG increase). Thus, carrying two lowering variants was protective against HTG (OR = 0.62; 95% CI, 0.39-0.98; *p *= 0.042) and having one single raising polymorphism (OR = 1.20; 95% CI, 1.39-2.87; *p *< 0.001) or more (2 or 3 raising variants; OR = 2.90; 95% CI, 1.56-5.41; *p *< 0.001) were associated with HTG.

**Conclusion:**

Our results showed a significant independent additive effect on TG levels of the *LPL *polymorphisms HindIII, S447X, D9N and N291S; the S19W and -1131T/C variants of *APOA5*, and the ε4 allele of *APOE *in our study population. Moreover, some of the variant combinations studied were significantly associated with the absence or the presence of hypertriglyceridemia.

## Background

The underlying determinants of individual susceptibility to cardiovascular disease (CVD) form a complex network of interactions between genetic and environmental risk factors, as a consequence of their multi-factorial nature [1]. Changes in triglyceride (TG) levels are now considered an independent cardiovascular risk factor [2,3]; hence, the study of combined variants in genes involved in TG metabolism may help explain part of the risk for CVD [4].

The influence of *LPL*, *APOA5 *and *APOE *genes on TG metabolism is well established. These genes code for proteins which are simultaneously present during the lipolysis of the TG core of circulating chylomicrons and VLDL. Lipoprotein lipase is the major TG-hydrolyzing enzyme [5], apolipoprotein AV has emerged as a regulator of TG levels by improving lipolysis efficiency [6-8] and apolipoprotein E, in addition to its critical role in receptor-mediated remnant clearance (liver uptake of TG-rich lipoproteins), is directly involved in lipolysis [9,10].

Numerous sequence variants have been described in all three of these genes. In *LPL*, D9N and N291S induce amino acid changes leading to lower post-heparin plasma LPL activity, affecting enzyme secretion or destabilizing homodimer complex formation, respectively. Both polymorphisms have been associated with increased plasma TG concentrations and with ischemic heart disease [11,12]. Inversely, the HindIII variant, which seems to be located within a regulatory element in intron 8 of the *LPL *gene [13], has been related with a protective effect [14]. Moreover, the nonsense polymorphism S447X has been associated with a gain of activity because of the premature truncation of the enzyme [15]. Consequently, it has also been related with lower fasting TG levels [14] and, very recently, with a favourable influence on the longitudinal changes of these levels [16]. The most informative polymorphisms within the *APOA5 *gene are the S19W missense polymorphism and the -1131T/C promoter variant, which define the initially described *APOA5 *haplotypes [17]. Both these polymorphisms have been associated with changes in TG levels in many populations, even with severe hypertriglyceridemia (HTG), as well as with CVD [18]. Genetic associations between the common *APOE *gene alleles, ε2, ε3 and ε4, and susceptibility to CVD and changes in cholesterol levels have been well replicated; however, disagreement still exists concerning *APOE *polymorphism and changes in TG concentrations [19].

The effects of each common variant involved in the expression of a multi-factorial trait are assumed to be modest and depend, at least in part, on differences in the environmental and the genetic context within the variants analyzed [20]. Therefore, the joint analysis of common functional variants of these genes, which have so far only been studied separately in many populations, might be important to better explain changes in TG levels.

We therefore determined the allele frequencies of four *LPL *variants (HindIII, S447X, D9N and N291S), two *APOA5 *variants (S19W and -1131T/C) and the *APOE *polymorphism (alleles ε2, ε3, ε4) in a large Mediterranean working population and assessed their combined effect on TG levels. Additionally, we studied the association between the presence of variant combinations (TG-lowering or TG-raising, or both) and TG levels and HTG.

## Methods

### Subjects

This work forms part of Ibermutuamur's Cardiovascular Risk Prevention Project [21,22] in which Spanish active workers, insured by this mutual insurance fund, were screened for cardiovascular risk factors at their annual medical check-up (MCU). The study conformed to the current Helsinki declaration and was reviewed and approved by Ibermutuamur's scientific Ethics Committee. The data presented here correspond to 1825 workers (1460 men and 365 women) from Malaga, a city on the Mediterranean coast of southern Spain, who attended for their routine MCU between 2004 and 2005 and gave written informed consent to participation in the study. The exclusion criteria were to be on sick leave and not signing the informed consent.

All the participants provided information about their date of birth, gender, profession, medication, smoking and alcohol consumption. Data on risk factors (diabetes, hypertension and cardiovascular diseases) were captured by medical record assessment following previously defined criteria [21]. The MCU included data on weight, height, waist circumference and blood pressure, as described [21]. Biochemical analyses were made from 12-hour fasting serum samples in Ibermutuamur's reference laboratories, using standard protocols. Participants were considered to be smokers if they smoked at the time of data collection or had quit smoking less than one year before. Regarding alcohol consumption, they were categorized as daily users versus occasional or non users. HTG was considered to be present if the fasting serum triglycerides ≥ 1.69 mmol/L [23].

### DNA extraction and genotyping

Genomic DNA was isolated from frozen EDTA whole blood using a BioRobot^® ^EZ1 (QIAGEN, Hilden, Germany) with the corresponding reagents. All PCR reactions needed for genotyping were carried out in a thermal cycler iCycler iQ™ (BioRad, California, USA). *APOE *(rs429358, rs7412) and Hind*III *(rs320) genotypes were determined by PCR and restriction fragment analysis using the iQ™ SYBR Green Supermix. The *APOE *amplification products were digested with HhaI following the manufacturer's instructions (Takara, Japan) and the resulting fragments separated on 4% agarose gel. Fragments corresponding to samples initially genotyped as ε2 carriers were also separated on 8% polyacrylamide gels to clearly distinguish between ε2ε2 and ε2ε3 genotypes. The digestion products obtained for the HindIII polymorphism were separated on 3.5% agarose gels. *LPL *(S447X -rs328-, N291S -rs268- and D9N -rs1801177-) and *APOA5 *(S19W -rs3135506- and -1131T/C -rs662799-) variants were determined by TaqMan assays using the iQ™ Supermix and the allele discrimination mode of the iQ™ software. The primer and probe sequences as well as the thermal protocols used in the PCR reactions are presented in the Additional file 1. The quality of genotyping was controlled using DNA samples of known genotypes, retyping those samples with an ambiguous band pattern on the same assay (for *APOE *and HindIII polymorphisms) and those with no interpretable optical PCR data (TaqMan assays) using PCR and restriction, and duplicating genotypes in 10% of randomly selected samples. The genotype error rate was estimated to be <1%.

### Statistical analyses

The statistical analyses were done using SPSS, version 14.0 for Windows and R [24]. A *p *value < 0.05 was considered statistically significant. Allele frequencies were calculated by direct counting. The SNPassoc package from R [25] was used to obtain the exact *p *values to verify Hardy-Weinberg equilibrium, to obtain measures of linkage disequilibrium and to check the validity of classifying individuals as carriers (heterozygous and homozygous for the minor allele) or non-carriers (homozygous for the frequent alleles) of each genetic variant that implies dominance between alleles. The TG variable was log-transformed to improve normality. The results were finally expressed in the original scale, after exponentiation of the adjusted least-square means, as geometric means and the corresponding asymmetric 95% confidence intervals. The ANOVA or the Student *t *tests were used to compare log-TG means between groups. Differences between genders were assessed using the Student *t *test to compare means of continuous variables, the Mann-Whitney U test to compare ordinal variables and the χ^2 ^test to compare percentages. When the expected values in more than 20% of cells were less than 5, Fisher's exact test was applied. A multivariate linear regression model with dummy variables for categorical terms was elaborated to test the null hypothesis of no association between each genetic variant assessed and log-TG, adjusting for the covariates: gender, age, waist circumference, blood glucose, blood pressure, smoking and alcohol consumption. Because of this logarithmic transformation, B coefficients associated with variant effects were multiplicative. We assessed, one at time, the significance of the two-factor interaction terms between variants. The critical *p *value after Bonferroni correction for multiple comparisons was 0.003. Logistic regression models were constructed to calculate the odds ratios for developing HTG associated with the presence of TG-lowering or TG-raising variants with respect to having no altering alleles (homozygous for the frequent alleles), adjusting for covariates. Standard regression diagnostic methods such as multicolinearity tests, the homogeneity of variance test and normal plots of the residuals were used to ensure the appropriateness of the models.

## Results

The main features of the study population are shown in Table 1. As found in the whole ICARIA study [21], men represented the majority of subjects (80%) and differed from women in the main biochemical and clinical variables analyzed. The prevalence of HTG (TG ≥ 1.69 mmol/L) was 22.8% (26.9% in men, 6.6% in women); only one man had TG > 11.29 mmol/L.

**Table 1:** Anthropometric, biochemical and life-style features of the study subjects.

	Total (n = 1825)	Women (n = 365)	Men (n = 1460)	^4^ *p*
Age (years)^1^	36 ± 10	34 ± 8	37 ± 10	<0.001
BMI (Kg/m^2^)^1^	27.0 ± 5.0	24.5 ± 5.0	28.6 ± 4.7	<0.001
Waist circumference (cm)^1^	92.1 ± 12.3	82.1 ± 11.0	94.3 ± 11.2	<0.001
Total cholesterol (mmol/L)^1^	5.16 ± 1.06	5.01 ± 0.89	5.20 ± 1.09	0.001
LDL cholesterol (mmol/L)^1^	3.27 ± 0.87	3.11 ± 0.74	3.31 ± 0.90	<0.001
HDL cholesterol (mmol/L)^1^	1.28 ± 0.31	1.48 ± 0.32	1.23 ± 0.28	<0.001
Non-HDL cholesterol (mmol/L)^1^	3.88 ± 1.01	3.53 ± 0.82	3.97 ± 1.04	<0.001
TG (mmol/L)^2^	1.12 (1.09-1.15)	0.83 (0.79-0.87)	1.21 (1.17-1.24)	<0.001
Blood glucose (mmol/L)^1^	4.88 ± 1.17	4.50 ± 0.50	5.00 ± 1.22	<0.001
Systolic blood pressure (mmHg)^1^	126 ± 17	113 ± 13	129 ± 16	<0.001
Diastolic blood pressure (mmHg)^1^	75 ± 12	71 ± 9	75 ± 12	<0.001
Smokers^3^	889 (49%)	158 (43%)	731 (50%)	0.015
Alcohol consumption^3^	326 (18%)	13 (3.6%)	313 (21%)	<0.001
Type 2 diabetes mellitus^3^	22 (1.2%)	1 (0.3%)	21 (1.4%)	0.102
CVD^3^	9 (0.56%)	0 (0%)	9 (0.7%)	0.369
Lipid lowering agent users^3^	28 (1.6%)	2 (0.6%)	26 (1.8%)	0.091
Cardiovascular risk SCORE^3^				<0.001
Low	1647 (94.2%)	350 (98.9%)	1297 (93%)	
Medium	15 (0.9%)	0 (0%)	15 (1.1%)	
High	86 (4.9%)	4 (1.1%)	82 (5.9%)	
Type of employement^3^				<0.001
Agriculture	6 (0.3%)	0 (0%)	6 (0.4%)	
Manufacturing	173 (9.8%)	29 (8.4%)	144 (10.2%)	
Construction	684 (38.8%)	55 (15.9%)	629 (44.4%)	
Services	902 (51.1%)	263 (75.8%)	639 (45.1%)	

^1 ^Variables expressed as means ± standard deviations.

^2 ^Variable expressed as geometric mean and 95% confidence interval

^3 ^Variables expressed as number of individuals and percentages.

^4 ^*p *value for the comparison between women and men.

LDL-C was calculated by the Friedewald formula for samples with TG < 4.52 mmol/L (n = 1799)

### Allele frequencies

All subjects were genotyped for the polymorphisms summarized in the text. Table 2 shows the genotype distribution and allele frequencies. Our population was in Hardy-Weinberg equilibrium for all variants analyzed. The highest frequencies of the rare variants of polymorphisms corresponded to the *LPL*-HindIII H- allele (29%) and to *LPL*-447X (12%) and the lowest frequencies to the *LPL*-9N (1.6%) and 291S (1.5%) alleles. The two *APOA5 *variants had similar allele frequencies (19W, 6.8%; -1131C, 6.2%) and the *APOE *alleles were, from the most to least frequent, ε3, ε4 and ε2, respectively. We found a strong linkage disequilibrium between the *LPL*-HindIII and S447X polymorphisms (D'= 0.984, r^2 ^= 0.308, *P *< 0.0001).

**Table 2:** Genotype distribution and allele frequencies of the *LPL*, *APOA5 *and *APOE *variants.

Gene	Variant	Reference sequence	Genotype distribution n (%)			Allele frequencies		*p* ^1^
*LPL*	Hind III	rs320	H+H+	920	(50.41)	H+	0.708	0.749
			H+H-	743	(40.71)	H-	0.292	
			H-H-	162	(8.88)			
								
*LPL*	S447X	rs328	447SS	1431	(78.41)	447S	0.884	0.470
			447SX	364	(19.95)	447X	0.116	
			447XX	30	(1.64)			
								
*LPL*	D9N	rs1801177	9DD	1768	(96.88)	9D	0.984	0.722
			9DN	56	(3.07)	9N	0.016	
			9NN	1	(0.05)			
								
*LPL*	N291S	rs268	291NN	1771	(97.04)	291N	0.985	0.892
			291NS	54	(2.96)	291S	0.015	
			291SS	0	(0.00)			
								
*APOA5*	S19W	rs3135506	19SS	1585	(86.85)	19S	0.932	0.982
			19SW	231	(12.66)	19W	0.068	
			WW	9	(0.49)			
								
*APOA5*	-1131T/C	rs662799	-1131TT	1609	(88.16)	-1131T	0.938	0.479
			-1131TC	206	(11.29)	-1131C	0.062	
			-1131CC	10	(0.55)			
								
*APOE*	C112R	rs429358	ε2ε2	4	(0.22)	ε2	0.057	0.741
	R158C	rs7412	ε2ε3	181	(9.92)	ε3	0.847	
			ε3ε3	1312	(71.89)	ε4	0.096	
			ε3ε4	287	(15.73)			
			ε4ε4	22	(1.21)			
			ε2ε4	19	(1.04)			

^1^Exact *p *value for Hardy-Weinberg equilibrium

### Associations between gene variants and TG levels

Table 3 shows TG geometric means and 95% confidence intervals (CI) according to the genotypes of the polymorphisms analyzed. These results confirm the lowering effect on TG levels of *LPL*-HindIII and S447X polymorphisms. In contrast, the presence of *LPL*-D9N, and the *APOA5 *-S19W and -1131T/C variants was associated with higher TG concentrations. Carriers of the *LPL*-N291S polymorphism showed a non-significant trend for higher levels of TG than non-carriers. Moreover, significant differences were also seen in TG levels between the *APOE *genotypes.

**Table 3:** Triglyceride geometric means and 95% confidence intervals (mmol/L) by *LPL*, *APOA5 *and *APOE *genotypes.

*LPL*-S447X^1^	447SS	1.15 (1.12-1.19)	*APOA5*-S19W^4^	19SS	1.11 (1.07-1.14)
	447SX	1.02 (0.96-1.07)^6^		19SW	1.19 (1.10-1.28)
	447XX	0.90 (0.75-1.10)		19WW	1.92 (0.94-3.96)^9^
					
*LPL*-HindIII^1^	H+H+	1.17 (1.13-1.22)	*APOA5*- (-1131T/C)^5^	-1131TT	1.11 (1.07-1.14)
	H+H-	1.07 (1.03-1.11)^7^		-1131TC	1.24 (1.15-1.33)
	H-H-	1.04 (0.97-1.13)^8^		-1131CC	1.39 (0.98-1.95)^10^
					
*LPL*-D9N^2^	9DD	1.12 (1.08-1.14)	*APOE*^1^	ε2ε2	0.62 (0.35-1.10)
	9DN	1.25 (1.07-1.47)		ε2 ε3	1.19 (1.03-1.29)
	9NN	3.56		ε3ε3	1.08 (1.05-1.12)
					
*LPL*-N291S^3^	291NN	1.12 (1.08-1.14)		ε3ε4	1.22 (1.14-1.30)^11^
	291NS	1.28 (1.11-1.46)		ε4ε4	1.50 (1.17-1.92)
	291SS	-		ε2ε4	1.00 (0.80-1.26)

Significant differences of ANOVA or the Student *t *test using log transformed variable (^1^*p *< 0.001; ^2^*p *= 0.043; ^3^*p *= 0.080; ^4^*p *= 0.005; ^5^*P *= 0.012).

Significant differences (*p *< 0.05) of post-hoc Bonferroni analysis: ^6^compared with 447SS; ^7,8^compared with H+H+; ^9^compared with 19SS and 19SW; one man TG>11.29 mmol/L; ^10^compared with -1131TT; ^11^compared with ε3ε3.

To elaborate the linear regression model, subjects were classified as carriers and non-carriers of the rare allele. In the case of *APOE *polymorphism, we grouped individuals as ε4 carriers (ε4ε4, ε3ε4 and ε2ε4) versus non-carriers and ε2 carriers (ε2ε2, ε2ε3 and ε2ε4) versus non-carriers. The ε4 carriers showed significantly higher TG levels (geometric mean 1.24 mmol/L; 95% CI: 1.16-1.32) than non-carriers (geometric mean 1.11 mg/dL; 95% CI: 1.06-1.13; *P *= 0.001). However, the ε2 carriers showed no significant differences in TG levels as compared with non-carriers. Similar results were obtained after excluding ε2ε4 subjects.

In order to avoid co-linearity in regression analyses, individuals with the *LPL*-HindIII and S447X polymorphisms were classified into three groups: 1) those who did not carry either HindIII or S447X (no protective variant), 2) carriers of either HindIII or S447X (one protective variant) and 3) carriers of both polymorphisms (two protective variants). The genotypes included in each group as well as their corresponding TG geometric means and the 95% confidence intervals are specified in Table 4. As can be seen, 99% of the subjects with one protective variant were carriers of the HindIII H- rare allele but not the 447X.

**Table 4:** Genotype distribution according to the presence of *LPL *HindIII and/or S447X polymorphisms.

Protective variants	Genotypes	TG^1^
No (N = 916)	H+H+447SS (n = 916)	1.17 (1.13 - 1.22)

One (N = 519)	H+H-447SS (n = 459)	1.11 (1.06 - 1.16)
	H-H-447SS (n = 56)	
	H+H+447SX (n = 4)	

Two (N = 390)	H+H-447SX (n = 284)	1.00 (0.95 - 1.06)^2^
	H-H-447SX (n = 76)	
	H-H-447XX (n = 30)	

H+ represents the most frequent allele of the HindIII variant and H- the least frequent allele. ^1^Geometric means (mmol/L) and 95% confidence intervals. *p *< 0.0001 of ANOVA test. ^2^Significantly different as compared to groups carrying one or no protective variant.

As shown in Table 5, after adjustment for the covariates (gender, age, waist circumference, blood glucose, blood pressure, smoking and alcohol use) the multiple regression model confirmed a significant TG-lowering effect of combined polymorphisms previously considered protective (when dummy variables evaluating HindIII or S447X separately were introduced in the model both reached statistical significance). Moreover, in our population, the *LPL*-D9N and *LPL*-N291S, and the *APOA5*-S19W, *APOA5*- (-1131T/C) variants and the *APOE*-ε4 allele were significantly associated with an independent additive TG-raising effect. Of all the variants analyzed, only the *APOE*-ε2 allele was not associated with changes in TG levels.

**Table 5:** Multiple linear regression model for the association between variants and TG levels adjusting for covariates.

Variable	B^1^	95% confidence interval	*p *value
*LPL*-protective variant combination^2^	0.920	0.895 - 0.947	<0.0001
*LPL*-D9N^3^	1.142	1.000 - 1.303	0.048
*LPL*-N291S^3^	1.198	1.048 - 1.369	<0.01
*APOA5*-S19W^3^	1.128	1.054 - 1.206	<0.001
*APOA5*- (-1131T/C)^3^	1.154	1.075 - 1.239	<0.0001
*APOE*-ε2^3^	1.038	0.966 - 1.115	0.309
*APOE*-ε4^3^	1.109	1.045 - 1.176	<0.001

Statistical analysis was performed excluding one subject with TG > 11.29 mmol/L and those taking lipid lowering drugs (n = 1731). R^2 ^of the model 0.30.

^1^Multiplicative coefficient of the effect of each variant, obtained after undoing logarithmic transformation.

^2^One protective variant or two protective variants versus reference (no protective variants).

^3^Carriers of the minor allele (homozygous and heterozygous) versus reference (homozygous for the frequent allele)

As no significant heterogeneity of the effect of any variant was detected according to gender, the remainder of the analyses were done for the group as a whole. Moreover, the corresponding interaction terms between variants were all far from reaching statistical significance.

### Analysis of variant combinations

The joint analysis of the polymorphisms revealed the presence of 46 different variant combinations, shown in the Additional file 2. Taking into account the low frequency of some of these and considering that the linear regression model indicated an independent effect of the polymorphisms, the subjects were grouped according to the presence of TG-lowering (L) and/or TG-raising (R) variants, as follows: (a) carriers of both HindIII and S447X (2L); (b) carriers of HindIII and S447X polymorphisms and any TG-raising variant (2L and R); (c) carriers of HindIII or S447X (1L); (d) subjects homozygous for the frequent alleles of the polymorphisms (No variant, NV); (e) carriers of one protective variant and any TG-raising polymorphism (1L and R); (f) carriers of one single TG-raising variant (1R), and (g) carriers of combinations of two or three TG-raising polymorphisms (2-3R). Due to their genotype frequencies (Table 4), 99% of the subjects in groups (c) and (e) were carriers of the HindIII polymorphism. No significant association was found between any of the seven groups and waist circumference, glycaemia, blood pressure, age, gender, smoking or alcohol consumption.

**Figure 1:**
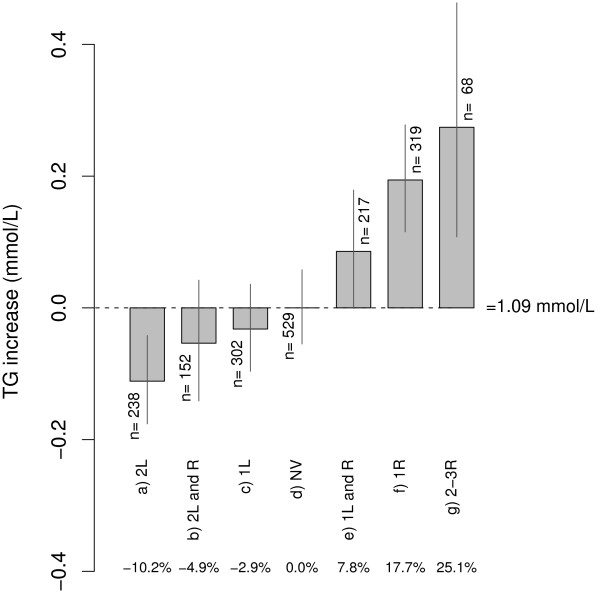
**Differences in TG geometric means and 95% confidence intervals for the groups of variant combinations**. The absolute values of the differences with respect to the NV group (no variant, i.e. homozygous for the frequent alleles of the polymorphisms) are given in mmol/L. The differences are expressed as percentages at the bottom of the figure. L: TG-lowering; R: TG-raising. *p *< 0.0001 for the mean comparisons using the ANOVA test.

**Figure 2:**
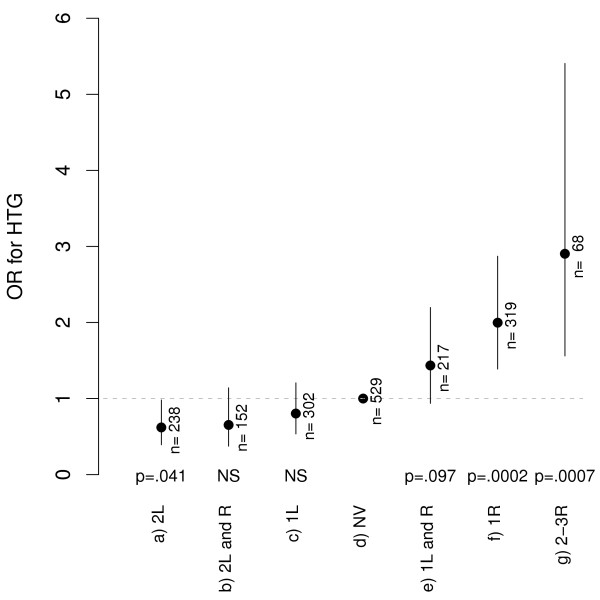
**Odds Ratios for hypertriglyceridaemia associated with the variant combinations**. NV: no variant, considered as the reference group. L: TG-lowering; R: TG-raising. HTG defined as TG levels ≥ 1.69 mmol/L.

Figure 1 shows the differences in TG levels of the variant combinations with respect to the "no variant" group, considered as the reference group. The greatest differences corresponded to carriers of two protective variants, with a 10.2% reduction in the TG geometric mean, and to carriers of two or three TG-raising polymorphisms, with a 25.1% increase in TG. Regarding the odds ratios (OR) for HTG (Figure 2), the presence of two TG-lowering polymorphisms was significantly associated with a protective effect (2L; OR = 0.62; 95% CI, 0.39-0.98, *p *= 0.042). Conversely, groups of single TG-raising variants (1R; OR = 1.20; 95% CI, 1.39-2.87, *p *< 0.001) or more than one TG-raising polymorphisms (2-3R; OR = 2.90; 95% CI, 1.56-5.41, *p *< 0.001) were significantly associated with HTG. This association disappeared in combinations with protective variants (non significant OR for groups "2L and R" and "1L and R").

## Discussion

Our study describes for the first time the simultaneous analysis of the genetic variants HindIII, S447X, D9N and N291S (*LPL*), S19W and -1131T/C (*APOA5*) and the *APOE *polymorphism and their association with TG levels in a large, well-characterized Spanish Mediterranean population. The results clearly show an independent effect of the polymorphisms studied, TG-lowering in the case of HindIII and S447X and with a clear TG-raising effect in the case of the D9N, N291S, S19W and -1131T/C variants and the APOε4 allele. Thus, combinations of lowering and/or raising polymorphisms showed a TG gradient below or above the TG levels of the reference (no variant) group. Moreover, the variant combination consisting of two lowering polymorphisms was protective against HTG, whereas having single or combined TG-raising variants was independently associated with HTG.

The frequency of carriers of *LPL*-D9N and N291S polymorphisms was around 3%, i.e., between the 1% and 7% described for healthy Caucasian persons [26,27], though no studies have, as far as we are aware, been carried out in Mediterranean populations of this size. We have only been able to find one Mediterranean study, that of Choumeriannou *et al*. [28], which showed a similar frequency (3.6%) for the *LPL*-N291S variant in a group of 84 Greek patients with familial hypercholesterolaemia. We have found no population studies in our area with which to compare the frequency of the D9N polymorphism and the allele frequency data presented is therefore original for this variant. The minor allele frequencies of *APOA5*- S19W and -1131T/C variants (6.2% and 6.8%, respectively) were similar to those of Caucasians elsewhere [17,29] and similar to those recently described in the Spanish EPIGEM population [30]. The protective *LPL *variants HindIII and S447X, and the *APOE *polymorphism have been studied in our country [14,31] and were found to be within the range reported for other populations from southern Europe [32,33].

Our work confirms the associations found in previous studies between the gene variants studied and TG levels [12,18,27,31], though it is the first time they have been analyzed simultaneously and in a large population, thereby showing their individual independent effect. Assuming a modest role of single polymorphisms as markers of complex diseases the study of variant combinations provides complementary information that could be clinically meaningful. Recent studies on genetic predisposition to CVD reinforce this idea as they show that certain polymorphism combinations, including variants in genes analyzed in our work, can predict CVD [4,34].

The results of our study show that the most favourable variant combination consisted of the presence of just TG-lowering variants of *LPL *and the least favourable was the presence, with no protective alleles, of at least one TG-raising variant. These results are in agreement with those of previous studies analyzing combinations of *LPL *variants [12] or combinations of *LPL *and *APOC3 *[14]. Additionally, our data suggest that the protective effect of HindIII and S447X is retained, not only versus the variants of *LPL *[12] but also versus those of *APOA5 *and *APOE*, which is a novel aspect of our study.

A recent report dealing with a Czech cohort forming part of the MONICA study [35] found that the raising effect of the *APOA5 *variants (-1131T/C or S19W) was significant in females but in males a non-significant trend was observed. Moreover, *APOA5 *did not affect plasma TG levels if the *APOE*-ε4 allele was present. Our results differ, as we clearly found an additive and independent effect of these variants of *APOA5 *and the other polymorphisms studied, with no heterogeneity according to gender. The lack of effect described by Hubacek *et al *is controversial, as previous results from the same group showed an increased frequency of the *APOA5*-19W*APOE-ε4 combination in persons with severe HTG as compared with a healthy population [36]. Sousa *et al *[37] have also described an interaction for the risk of developing severe HTG associated with the combination *APOA5*-1131C*APOE-ε2. In our study, however, we did not find a main TG-raising effect associated with the *APOE*-ε2 allele; conversely, this lack of association with TG levels has been described in other populations for the *APOE*-ε4 allele [38,39]. On other hand, the higher TG levels seen by us in APOA5*APOE and other variant combinations were not significantly associated with an interaction effect. It is important to note the differences between these studies regarding their design (case-control, population-based,...) and in terms of the statistical approach (univariate or regression analyses, different covariates used,...), and there may also be situations of limited statistical power. Finally, a major consideration is that the differences in the environmental and genetic context of the populations studied may strongly influence the results obtained and thus explain the lack of concordance among these studies.

As expected, the presence of a TG-raising polymorphism, and even more so that of a combination of these variants, was associated with a greater likelihood of HTG, as Figure 2 clearly shows. Prior analyses by another group [38] have shown an increase in the OR with effect from two TG-raising variants, depending on the waist circumference; indeed, the effect was amplified when the number of TG-raising polymorphisms rose together with the presence of abdominal obesity. This contrasts with data from the present study, since the association with HTG with effect from one TG-raising variant was independent of waist circumference. The combined analysis of the TG-lowering and TG-raising variants, which is original to this study, partly explains these differences, as the TG-lowering polymorphisms were not analysed in the study by Brisson *et al*. As can be seen in Figure 2, the association of the TG-raising variants with HTG lost its significance, even with just one protective variant, i.e. with the *LPL*- HindIII intronic polymorphism. Therefore, our results reinforce the concept that this polymorphism has a modestly advantageous effect independent of the S447X variant (in linkage disequilibrium with it and classically considered functional) and highlight the importance of analyzing both protective variants.

Finally, the association of the *APOA5 *polymorphisms S19W and -1131T/C, and the *APOE *non-ε3 genotype with severe HTG has been recently reported [40]. This report also highlights the multiplicative effect in the value of the Odds Ratios for severe HTG of combinations of six TG-raising polymorphisms, always with one of the *APOA5 *variants. Unfortunately, despite the fact that our study population was large, the relatively small number of persons with TG-raising combinations and with HTG limits the statistical power to detect certain potential interactions between variants.

Although our study supports previous results from other groups and provides new findings with consistent results, certain limitations are involved. Firstly, it is obvious that the characteristics inherent to the study population (geographical area, age, state of health) condition the results obtained. Secondly, analysis of more variants in more genes as well as other environmental variables [41], such as diet and physical activity, not assessed in our study might likely better explain variability in TG levels and the manifestation of HTG.

## Conclusion

This work, carried out in a subpopulation of 1825 subjects of the ICARIA study, revealed an independent and additive effect of the polymorphisms HindIII, S447X, D9N and N291S of *LPL*, S19W and -1131T/C of *APOA5 *and the ε4 allele of *APOE *on TG levels. Combinations of TG-lowering (HindIII/S447X) and/or TG-raising variants (the other polymorphisms) were related to different TG levels and, as a consequence, some of these variant combinations were significantly associated with the absence (2 TG-lowering polymorphisms) or the presence (one or more TG-raising variants) of HTG. Because serum TG are markedly associated with vascular disease in the ICARIA population [3], the follow-up of our cohort may shed light on the association between genetic variant combinations and new vascular disease.

## Competing interests

A non-financial competing interest is declared, related to the genotyping work, as three patent applications have been applied for:

• Ariza MJ, Rioja J, Valdivielso P, Sánchez-Chaparro MA and González-Santos P. Set of primers, probes, procedure and kit for the genotyping of the *APOA5 *genetic polymorphism S19W. N°: P200802183.

• Ariza MJ, Rioja J, Valdivielso P, Sánchez-Chaparro MA and González-Santos P. Set of primers, probes, procedure and kit for the genotyping of the APOA5 genetic polymorphism -1131T/C. N°: P200802184.

• Ariza MJ, Rioja J, Valdivielso P, Sánchez-Chaparro MA and González-Santos P. Set of primers, probes, procedure and kit for the genotyping of the LPL genetic polymorphism S447X. N°: P200802182.

## Authors' contributions

MJA coordinated and participated in genotyping, participated in the analysis of the data and wrote the first draft. MASC coordinated the scientific design of the ICARIA project and the local recruitment of subjects and clinical data capture. FJB conducted the statistical analyses and elaborated the vectorial format of figures. AMH carried out most of the practical work (sample processing, DNA extraction and genotyping). ECB coordinated the computer and statistical applications of the ICARIA project. JAG, coordinated the medical teams of the ICARIA project. ECB and JAG assisted in the recruitment of subjects and clinical data capture. JR assisted in practical work and provided critical revisions of the manuscript. PV revised the manuscript critically for important intellectual content; PGS conceived, designed and coordinated the study. All authors have contributed to and approved the final version of the manuscript.

## Acknowledgements

This work was partially supported by a grant of the Spanish Atherosclerosis Society (Beca FEA/SEA 2006 para Investigación Clínico-Epidemiológica) and by the Andalusian Government (Ayuda de Investigación a la Unidad de Investigación de Lípidos y Ateriosclerosis CTS157 convocatoria 2007).

The authors would like to thank Guadalupe Requena-Santos (MD), Martha Cabrera-Sierra (MD), Juan Carlos Sáinz-Gutiérrez (MD) and Montserrat Ruiz-Moraga for their contribution in clinical data capture and management. We also thank Ian Johnstone for his help with the English language version of the manuscript.

## Pre-publication history

The pre-publication history for this paper can be accessed here:

http://www.biomedcentral.com/1471-2350/11/66/prepub

## Supplementary Material

Additional file 1 Primers and probes sequences and thermal cycling conditions used for DNA amplification and genotyping. Click here for file

Additional file 2 Variant combinations observed in the study population. Click here for file
