# Supplementary material for: Additive effects of LPL, APOA5 and APOE variant combinations on triglyceride levels and hypertriglyceridemia: results of the ICARIA genetic sub-study
Source: BMC Med Genet. 2010 Apr 29;11:66. doi: 10.1186/1471-2350-11-66 (PMC2877669; doi:10.1186/1471-2350-11-66)
Supplement: Additional file 1 — Primers and probes sequences and thermal cycling conditions used for DNA amplification and genotyping. [file 1471-2350-11-66-S1.DOC]

| Additive effects of *LPL*, *APOA5* and *APOE* variant combinations on triglyceride levels and hypertriglyceridemia: results of the ICARIA genetic sub-study. | | | | | | |
| --- | --- | --- | --- | --- | --- | --- |
| **Additional file 1** | | | | | | |
| **Primers and probes sequences and thermal cycling conditions used for DNA amplification and genotyping** | | | | | | |
| **Gene** | **Variant** | **ID** | **NC** | **Primer sequences (5’→3’)** | **Probe sequences (5’→3’)** | **Annealing/Amplification** |
| *LPL* | Hind III^a^ | rs320 | T/G | **F**: TTCCAAGATAATCTCACCCT | No | 30 s 55 °C, 30s 72°C |
|  |  |  |  | **R**: AACAGCACACTATAGTTTGC | No |  |
|  |  |  |  |  |  |  |
| *LPL* | S447X^b^ | rs328 | G/C | **F**: AGGAAAGGCACCTGCGGTA | F1- ATGCTCACCAGCCT**G**ACTTCTTATTCA-Q | 30 s 64 °C |
|  |  |  |  | **R**: CAGGATGCCCAGTCAGCTTT | F2- ATGCTCACCAGCCT**C**ACTTCTTATTCA-Q |  |
|  |  |  |  |  |  |  |
| *LPL* | D9N^b^ | rs1801177 | G/A | **F**: CTCCAGTTAACCTCATATCCAATT | F1-TTACTTTCGATGT**C**GATAAAATCTCTTCT-Q | 30 s 60 °C |
|  |  |  |  | **R**: GGTGGCAAGTGTCCTCAG | F2-ATTTACTTTCGATGT**T**GATAAAATCTCTTCT-Q |  |
|  |  |  |  |  |  |  |
| *LPL* | N291S^b^ | rs268 | A/G | **F**: GGCTCTGCTTGAGTTGTAGA | F1-TGGCTCTGACTTTA**T**TGATCTCATAGC-Q | 30 s 60 °C |
|  |  |  |  | **R**: AGAACGAGTCTTCAGGTACATT | F2-TTGGCTCTGACTTTA**C**TGATCTCATAG-Q |  |
|  |  |  |  |  |  |  |
| *APOA5* | S19W^b^ | rs3135506 | C/G | **F**: CCCTGATTACCTAGTCCCTCT | F1-ACAGCGTTTT**C**GGCCACCCA-Q | 30 s 64 °C |
|  |  |  |  | **R**: ATCTTCTGCTGATGGATCTGCT | F2-ACAGCGTTTT**G**GGCCACCCA-Q |  |
|  |  |  |  |  |  |  |
| *APOA5* | -1131T/C^b^ | rs662799 | T/C | **F**: CCTGCGAGTGGAGTTCAGCTTT | F1-ATGGGGCAAATCT**T**ACTTTCGCTCCA-Q | 30 s 68 °C |
|  |  |  |  | **R**: AACAAGCAAGGGAAGCCAGG | F2-ATGGGGCAAATCT**C**ACTTTCGCTCCA-Q |  |
|  |  |  |  |  |  |  |
| *APOE* | C112R^a^ | rs429358 | T/C | **F**: AGACGCGGGCACGGCTGTCCAA | No | 30 s 72 °C |
|  | R158C^a^ | rs7412 | C/T | **R**: GGATGGCGCTGAGGCCGCGCTC | No |  |
| ID: identification by reference sequence number. NC: nucleotide change. F: forward. R: reverse. The DNA was amplified in ^a^25 µL reaction volume containing 500 nM of each primer; ^b^15 µL reaction volume containing 300 nM of each primer, 100 nM of the more frequent allele specific probe, labeled at the 5’ end with Fam (6-carboxifluoresceine: F1) and 200 nM of the specific probe for the less frequent allele, labeled at the 5’ end with Hex (hexaclorofluoresceine: F2). Q: Tamra (carboxitetrametilrodhamine). All PCR reactions had an initial denaturation step of 5 minutes and 40 cycles with 30 seconds of denaturation and subsequently steps of annealing/amplification as indicated in the table for each polymorphism. | | | | | | |
