# Supplementary material for: Additive effects of LPL, APOA5 and APOE variant combinations on triglyceride levels and hypertriglyceridemia: results of the ICARIA genetic sub-study
Source: BMC Med Genet. 2010 Apr 29;11:66. doi: 10.1186/1471-2350-11-66 (PMC2877669; doi:10.1186/1471-2350-11-66)
Supplement: Additional file 2 — Variant combinations observed in the study population. [file 1471-2350-11-66-S2.DOC]

Additive effects of *LPL*, *APOA5* and *APOE* variant combinations on triglyceride levels and hypertriglyceridemia: results of the ICARIA genetic sub-study

| **Additional File 2** | | | | | | | | |
| --- | --- | --- | --- | --- | --- | --- | --- | --- |
| **Variant combinations observed in the study population** | | | | | | | | |
| Group | N | S447X | Hind III | D9N | N291S | S19W | -1131T/C | APOε4 |
| No variant | 529 | 0 | 0 | 0 | 0 | 0 | 0 | 0 |
| 2 L | 238 | **1** | **1** | 0 | 0 | 0 | 0 | 0 |
| 2 L and R (N=152) | 4 | **1** | **1** | **1** | 0 | 0 | 0 | 0 |
|  | 5 | **1** | **1** | 0 | **1** | 0 | 0 | 0 |
|  | 37 | **1** | **1** | 0 | 0 | **1** | 0 | 0 |
|  | 28 | **1** | **1** | 0 | 0 | 0 | **1** | 0 |
|  | 56 | **1** | **1** | 0 | 0 | 0 | 0 | **1** |
|  | 1 | **1** | **1** | **1** | 0 | **1** | 0 | 0 |
|  | 1 | **1** | **1** | 0 | **1** | 0 | **1** | **1** |
|  | 2 | **1** | **1** | 0 | **1** | **1** | 0 | 0 |
|  | 2 | **1** | **1** | 0 | 0 | **1** | **1** | 0 |
|  | 7 | **1** | **1** | 0 | 0 | **1** | 0 | **1** |
|  | 9 | **1** | **1** | 0 | 0 | 0 | **1** | **1** |
| 1 L (N=302) | 3 | **1** | 0 | 0 | 0 | 0 | 0 | 0 |
|  | 299 | 0 | **1** | 0 | 0 | 0 | 0 | 0 |
| 1 L and R (N=217) | 1 | **1** | 0 | 0 | 0 | **1** | 0 | 0 |
|  | 4 | 0 | **1** | **1** | 0 | 0 | 0 | 0 |
|  | 19 | 0 | **1** | 0 | **1** | 0 | 0 | 0 |
|  | 41 | 0 | **1** | 0 | 0 | **1** | 0 | 0 |
|  | 46 | 0 | **1** | 0 | 0 | 0 | **1** | 0 |
|  | 65 | 0 | **1** | 0 | 0 | 0 | 0 | **1** |
|  | 1 | 0 | **1** | **1** | **1** | 0 | 0 | 0 |
|  | 1 | 0 | **1** | **1** | **1** | 0 | 0 | **1** |
|  | 2 | 0 | **1** | **1** | 0 | **1** | 0 | 0 |
|  | 3 | 0 | **1** | **1** | 0 | 0 | 0 | **1** |
|  | 3 | 0 | **1** | 0 | **1** | 0 | **1** | 0 |
|  | 2 | 0 | **1** | 0 | **1** | 0 | 0 | **1** |
|  | 3 | 0 | **1** | 0 | 0 | **1** | **1** | 0 |
|  | 3 | 0 | **1** | 0 | 0 | **1** | **1** | **1** |
|  | 11 | 0 | **1** | 0 | 0 | **1** | 0 | **1** |
|  | 12 | 0 | **1** | 0 | 0 | 0 | **1** | **1** |
| 1 R (N=319) | 27 | 0 | 0 | **1** | 0 | 0 | 0 | 0 |
|  | 15 | 0 | 0 | 0 | **1** | 0 | 0 | 0 |
|  | 95 | 0 | 0 | 0 | 0 | **1** | 0 | 0 |
|  | 75 | 0 | 0 | 0 | 0 | 0 | **1** | 0 |
|  | 107 | 0 | 0 | 0 | 0 | 0 | 0 | **1** |
| 2-3 R (N=68) | 3 | 0 | 0 | **1** | 0 | **1** | 0 | 0 |
|  | 5 | 0 | 0 | **1** | 0 | 0 | **1** | 0 |
|  | 6 | 0 | 0 | **1** | 0 | 0 | 0 | **1** |
|  | 2 | 0 | 0 | 0 | **1** | **1** | 0 | 0 |
|  | 1 | 0 | 0 | 0 | **1** | 0 | **1** | 0 |
|  | 2 | 0 | 0 | 0 | **1** | 0 | **1** | **1** |
|  | 1 | 0 | 0 | 0 | 0 | **1** | **1** | **1** |
|  | 6 | 0 | 0 | 0 | 0 | **1** | **1** | 0 |
|  | 23 | 0 | 0 | 0 | 0 | **1** | 0 | **1** |
|  | 19 | 0 | 0 | 0 | 0 | 0 | **1** | **1** |
| N: number of subjects. “No variant” group: subjects homozygous for the more frequent alleles of the polymorphisms. L: TG-lowering; R: TG-raising. The presence of the least frequent allele of each polymorphism is represented by “1” and the absence by “0”. | | | | | | | | |
